# Type IA Oromandibular-Limb Hypogenesis Syndrome: A Case Report and A Case Update

**DOI:** 10.7759/cureus.24647

**Published:** 2022-05-01

**Authors:** Celine Richard, Amy Manning, Gregory Peason, Scott E Hickey, Andrew R Scott, Jonathan Grischkan

**Affiliations:** 1 Pediatric Otolaryngology, University of Tennessee Health Science Center, Memphis, USA; 2 Pediatric Otolaryngology, Nationwide Children’s Hospital, Columbus, USA; 3 Pediatric Plastic and Reconstructive Surgery, Nationwide Children’s Hospital, Columbus, USA; 4 Pediatrics, Section of Molecular and Human Genetics, Nationwide Children’s Hospital, Columbus, USA; 5 Pediatric Otolaryngology and Facial Plastic Surgery, Hospital for Children at Tufts Medical Center, Boston, USA; 6 Otolaryngology, Nationwide Children's Hospital, Columbus, USA; 7 Otolaryngology, Ohio State University Wexner Medical Center, Columbus, USA

**Keywords:** hypoglossia, micrognathia, transverse discrepancy, multidisciplinary approach, feeding disorders

## Abstract

Hypoglossia is a rare congenital anomaly resulting in a small rudimentary tongue. It is classified under the oromandibular-limb hypogenesis syndrome and can be found in isolation (Type IA) but is more often associated with other congenital disorders, such as limb defects. Isolated hypoglossia cases are rare, and while feeding disorders are common, in some cases, neonatal airway obstruction is the most problematic. In the present report, we discuss two cases of newborns presenting with hypoglossia without limb deformities or visceral anomalies: one new case and a 10-year update of a previously reported case. These two cases highlight the variability in presenting symptoms and the challenges in diagnosis and management of a rare clinical entity. We focus on the discussion of early diagnosis, multidisciplinary management, and shared decision-making, with emphasis on the current therapeutic strategies available to the clinician and their limitations during the neonatal period. Early surgical multivector mandibular distraction osteogenesis can be proposed with minimal short- and long-term morbidity, pending a consistent follow-up. This clinical entity will require multidisciplinary team care into adult years.

## Introduction

Isolated hypoglossia (also termed microglossia) [1] is a very rare congenital anomaly resulting in a “small rudimentary tongue.” The assessment of the tongue size is subjective and requires a careful examination both at rest and during motion [2,3]. This may partly explain why the exact frequency of hypoglossia among newborns is unknown. Over the last two centuries, fewer than 30 cases of isolated hypoglossia have been reported in the literature. Hypoglossia can be found in isolation but is most commonly associated with other congenital disorders, involving the oromandibular complex (e.g., cleft palate, dental malformations, and maxillomandibular attachment) or the limbs. Hall [1] compiled these different clinical presentations and classified them as part of the oromandibular-limb hypogenesis syndrome (OLHS, MIM 103300) in which the only criterion for inclusion is hypoglossia. Type I OLHS presents either as isolated hypoglossia (Type IA) or aglossia (Type IB) [1]. Despite the initial common anatomical presentations, the two cases presented herein (one with long-term follow-up [4]) illustrate the differences in clinical course experienced by infants and children with hypoglossia. Few reports exist in the literature on isolated micro/hypoglossia and even fewer with early surgical intervention.

## Case presentation

Case 1

Neonatal Case

**Figure 1:**
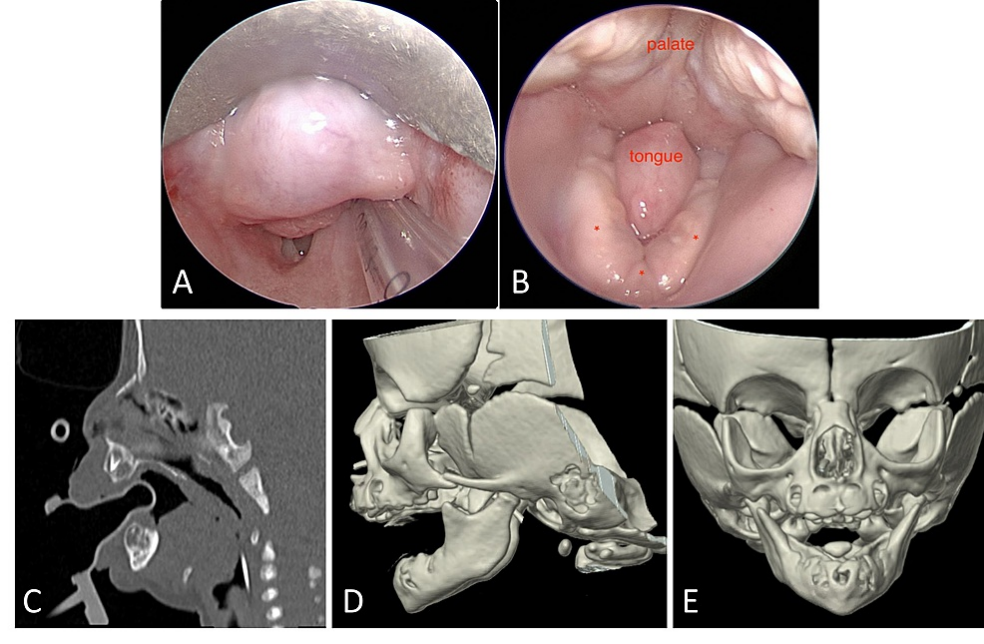
Case 1 (A) Short rudimentary tongue filling the narrow space between the right and left mandibular bodies. (B) Direct laryngoscopy revealing the glossoptosis and supraglottic obstruction. (C) Computed tomographic scan of the two-week-old infant in the sagittal plane with soft tissue window. Note the absent anterior tongue and its posterior part causing glossoptosis obstructing the upper airway. (D) 3D view of the hypoplastic mandible and condyles. (E) 3D view of the transverse deficiency.

The otolaryngology team was called to the bedside to evaluate a newborn presenting with increased work of breathing and requiring an increasingly higher flow of oxygen via nasal cannula. The infant was born at 42.2 weeks of gestational age to a 22-year-old gravida 1, para 1 woman. On examination, the infant had a high-arched palate, hypoglossia, micrognathia, glossoptosis (Figure 1, Panels A and B), transverse mandibular deficiency, and right eyelid ptosis. Although some of these features are part of the Pierre Robin sequence (PRS), association with hypoglossia and transverse deficiency are classified as Type IA birth defects per Hall’s classification of OLHS [1]. The infant’s prenatal course was significant for intrauterine growth retardation and marijuana exposure. All other prenatal screening laboratory bloodwork was negative.

The genetics consultation did not identify any underlying syndromic diagnosis. Echocardiogram, renal ultrasound, and chromosomal microarray were all normal. Early feeding and swallowing evaluation revealed normal gag, rooting, and sucking reflexes but an inability to actively extend the small rudimentary tongue. The infant was unable to manage her secretions or oral feeding bolus. Multidisciplinary discussions to determine the plan of care included members from neonatology, psychology, nutrition, speech therapy, genetics, otolaryngology, and craniofacial/plastic surgery. The infant remained dependent on nasogastric tube feeding and required continuous non-invasive ventilation with side positioning. Due to the shortened mandibular bodies and the V-shaped, narrow mandibular symphysis, the patient was deemed not a candidate for neonatal mandibular distraction osteogenesis (MDO). Ultimately, this necessitated tracheostomy tube placement, in conjunction with gastrostomy-tube (G-tube) placement at seven weeks of age. The child is enrolled in feeding intervention since birth to improve the oral motor function and strength. A pre-MDO planning maxillofacial computed tomography (CT) scan performed at six months of age revealed interval improvement in micrognathia. At this follow-up, she was also tolerating baby foods by mouth, taking full feeding via bottle, and minimally using the G-tube only for medications.

Case 2

A 10-Year Update of a Previously Reported Case

**Figure 2:**
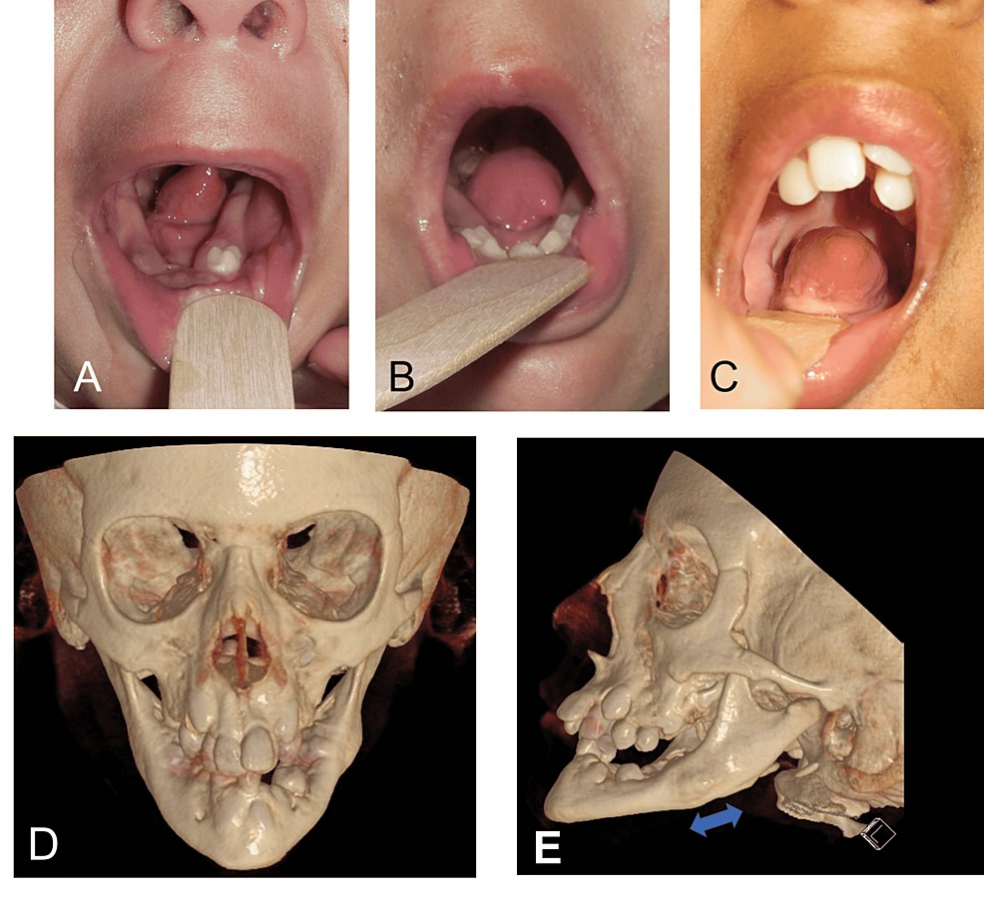
Case 2 (A) Microglossia of the anterior tongue with glossoptosis, at 16 months of age. (B) Correction of glossoptosis following mandibular distraction osteogenesis, at 18 months of age. (C) Sustained correction of oral tongue positioning, at 10 years of age. (D) 3D maxillofacial CT, at age 10, nine years after bilateral mandibular distraction osteogenesis using external multivector distractors. The left panel shows severe mandibular and maxillary constriction with telescopic bite. (E) The blue arrow indicates the region of regenerated bone.

A full-term infant presented with an isolated small anterior tongue. The child was noted to have severe feeding difficulty requiring a G-tube placement but no clinical signs of upper airway obstruction. At 16 months of age, the sleep study was normal, but the examination was notable for severe transverse constriction of the mandible and a small anterior 2/3 of the tongue with an otherwise normal tongue base. Given his severe feeding difficulties in the presence of glossoptosis (Figure 2, Panel A), bilateral MDO was proposed in an effort to improve the feeding difficulty and potentially positively impact speech as this would allow for positioning of the small anterior tongue into the oral cavity. The child underwent MDO with multivector external devices at 17 months of age. Correction of the glossoptosis was observed (Figure 2) with improvement in oral feeds. Removal of the MDO and gastrostomy tubes were successfully achieved three months later.

In early childhood, he struggled with articulation errors, given the limited range of motion of the hypoplastic anterior tongue. He also had significant sialorrhea, possible mechanisms of which included impaired tongue movement, narrow/obliterated floor of mouth related to transverse mandibular hypoplasia, or impaired mental nerve sensation following MDO. He received onabotulinumtoxin A with good effect every six months until approximately age 4, at which point symptoms had improved. He underwent adenotonsillectomy for sleep-disordered breathing symptoms and required regular dental restorations under anesthesia.

**Figure 3:**
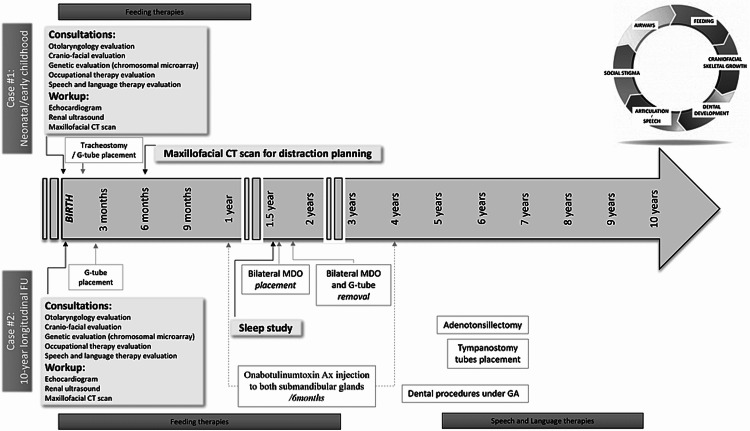
Workup and interventions timeline MDO: Mandibular distraction osteogenesis.

The child received early intervention, speech and feeding services, and a set of tympanostomy tubes. His eustachian tube dysfunction had resolved by age 5, and other than receiving speech therapy through the school system for articulation errors, he had no developmental concerns and remained in age-appropriate grade level throughout his education. Figure 3 shows workup and intervention timelines.

## Discussion

This report provides us with an insight into this rare clinical entity. Complete prenatal history and genetic microarray testing were negative for Case 2 and positive for marijuana exposure only in Case 1. While prenatal medication exposure (i.e., Diazepam and Tigan) [5] has been suggested to impact the development of the lingual and palatal structures between the fourth and eighth weeks of gestation [2,3], its causal relationship has not yet been proven. Given potentially associated malformations, early workup should include maxillofacial CT scan, renal ultrasound, chest x-ray, echocardiogram [6], brain ultrasound, and genetic evaluation as it is often pursued in patients with congenital anomalies of unknown etiology [1,7-16]. Speech and swallow evaluation with a potential video swallow study can help complete the initial assessment (Table 1).

**Table 1:** Review of relevant literature: early workup and findings Only cases reported as Type IA. WNL: Within normal limits; FEES: Fiberoptic endoscopic evaluation of swallowing; SLP: Speech and language therapy; CT scan: Computed tomography scan; CMP: Complete metabolic panel; DLB: Direct laryngobronchoscopy.

Isolated Microglossia										
Publication	Patient	Prenatal exposure	Micrognathia	Decreased transverse and AP mandibular dimensions	Glossoptosis	Palate abnormality	Airway obstruction	Initial workup	Swallow evaluation	Subsequent studies
Roth et al., 1972 [8]	#1	No	Yes	Yes	No	High-arched and constricted palate	No	CMP, thyroid testing, chest x-ray, IV pyelogram (all WNL)	SLP evaluation, Barium swallow study	No
Kuroda and Ohyama 1981 [11]	#1	No	Yes	Yes	No	Unspecified	None in infancy	Unknown	No	Longitudinal cephalometric study
Weingarten et al., 1993 [2]	#1	Marijuana, cigarette smoke, and alcohol	Yes	Yes	Yes	No	Yes	Brain and kidney US, DLB	Yes, at 1 month of age	Maxillofacial CT scan at 15 months
Yamada et al., 2000 [12]	#1	No	Yes	Yes	No	Submucosal cleft palate	Yes	Unknown	Unknown	Maxillofacial CT scan at 9 yo, taste examination (WNL)
	#2	No	Yes	Yes	Unknown	No	Yes	Unknown	Unknown	Maxillofacial CT scan at 9 yo
Thorp et al., 2003 [7]	#1	Unknown	Yes	Unknown	Unknown	Unknown	No	Unknown	Unknown	No
	#2	Alcohol exposure	Yes	Yes	Yes	No	Yes	DLB	SLP evaluation	Unknown
	#3	Unknown	Yes	Yes	Unknown	Maxillo-mandibular fibrous adhesions	Yes	Unspecified	SLP evaluation	Unknown
	#4	Unknown	Yes	Unknown	Unknown	Unspecified	Yes	Unspecified	SLP, Barium swallow	Unknown
	#5	Alcohol	Yes	Unknown	Unknown	Cleft palate	Yes	Unspecified	No	Unknown
Voigt et al., 2012 [4]	#1	Unknown	Yes	Yes	Yes	Submucous cleft palate	No	DLB, maxillofacial CT scan, chest X- ray	SLP evaluation, barium swallow study, and FEES	
Sharma et al., 2012 [9]	#1	No	Yes	Yes	Unknown	High-arched and constricted palate	No	Thyroid function	No	No
Noyola-Frias et al., 2013 [10]	#1	Second-hand exposure to marijuana and tobacco smoke	Yes	Unknown	Unknown	Shortened soft palate fused to tonsillar pillar	No	Limbs x rays, brain CT scan, CMP, and thyroid test (all WNL)	Yes, aspiration penumonia	No
Nepram et al., 2015 [13]	#1	No	Yes	Yes	No	Unspecified	No	Unspecified	No	No
Ogawa et al., 2015 [14]	#1	No	Yes	Yes	Unknown	No	Yes	Unknown	Unknown	panoramic radiograph
Gopal et al., 2017 [16]	#1	No	Hemimandibular hypoplasia	No	Unknown	Unspecified	No	MRI (Unspecified)		Unknown
Imai et al., 2019 (update from 1999) [15]	#1	No	Yes	Yes	No	Submucosal cleft palate	Yes	Unknown	Unknown	Maxillofacial CT scan at 9 yo, taste examination (WNL)
	#2	No	Yes	Yes	Unknown	No	Yes	Unknown	Unknown	Maxillofacial CT scan at 9 yo
	#3	No	Yes	Yes	Unknown	No	No	Unknown	Unknown	Unknown
Wallace et al., 2020 [18]	#2	Unknown	Yes	Yes	Yes	Unknown	Yes	Unknown	Unknown	Unknown

For some, polysomnography may be considered. Although both cases presented with hypoglossia, transverse mandibular deficiency, and glossoptosis, their neonatal course and intervention strategy differed. While for Case 1, the airway symptoms ultimately required tracheostomy tube placement, Case 2’s initial clinical presentation was dominated by feeding disorders. In our cases, the variation in tongue size and degree of glossoptosis were the main factors accounting for the difference in airway status.

A thorough assessment of the airways and swallowing function is critical for proper management. While direct laryngoscopy and bronchoscopy are the gold standards with regard to airways evaluation, a recent report highlighted the potential utility of ultrasonographic examination of the larynx in diagnosis (i.e., laryngeal abnormalities) and speech and swallowing function assessment [17]. This inexpensive, safe, and non-invasive procedure, that can be performed at the bedside, could be considered to assist clinicians in the decision-making strategy.

So far, the isolated microglossia cases described in the literature are presented with signs of PRS. As in any PRS case, the airway compromise can present early and require intervention. The first step in such cases is to opt for functional maneuvers aiming at relieving a part of the airway collapse. Prone or side-lying positioning and nasopharyngeal airways are the first steps and may allow for adequate ventilation in some cases. To counteract the impact of glossoptosis on the airway, surgical strategies may include tongue-lip adhesion, MDO, or tracheostomy (Table 2). Choice of one procedure over the others will be driven by both the patient’s anatomical and clinical status, the input from the different multidisciplinary healthcare specialists, and the caregivers’ preferences. However, contrary to patients with PRS in whom MDO may be considered within the first weeks after birth, the impaired mandibular development in hypoglossia/micrognathia cases may prevent the placement of a distraction device and can complicate the planned distraction vectors. In such a case with airway compromise, the early and safest option is deemed to be a tracheostomy tube placement, as done in Case 1. In both cases, the constricted mandible associated with hypoglossia limited the infants’ oral motor function and bolus propulsion abilities, mandating a nasogastric and then ultimately G-tube placement.

**Table 2:** Review of relevant literature: early interventions and short- and long-term follow-up VPI: Velopharyngeal insufficiency; NG tube: Nasogastric tube; G-tube: Gastrostomy tube.

Isolated Microglossia												
Publication	Patient	Feeding difficulties	Speech therapy required	Early NG tube placement	Tracheostomy	Age at tracheostomy	Age at decannulation	Age at NG tube removal	Other interventions	Speech and language		Age at last follow-up
Roth et al., 1972 [8]	#1	NG initially placed	No (normal sucking and swallowing)	Yes	No	NA	NA	Early infancy			Unknown	9 months
Kuroda and Ohyama 1981 [11]	#1	None in infancy	No	No	No	NA	NA	NA	Orthodontic intervention		Sounds distortion and phonemes substitution	8 years
Weingarten et al., 1993 [2]	#1	Some aspirations	Yes	No	Yes	Neonatal period	Unknown	NA			Reportedly normal	15 months
Yamada et al., 2000 [12]	#1	Yes	Yes	Yes	No	NA	NA	12 months	Mandibular distraction (linear), then orthodontic treatment at 9 years of age		Articulation errors	See Imai et al., (2019)
	#2	Yes	Yes	Yes	Yes	50 days	Unknown	3 years	Mandibular distraction (linear) at 9 years of age		Articulation errors	Unknown
Thorp et al., 2003 [7]	#1	Yes	Unknown	Yes	No	NA	NA	Unknown	No		Unknown	Unknown
	#2	Yes	Yes	Yes	Yes	Early infancy	30 months	17 months			Articulation difficulties, receptive, and expressive delays	30 months
	#3	Yes NG and then G-tube	Yes	Yes	Yes	Neonatal	NA	NA	Coronal osteotomies and adhesions release at 14 months		Unknown	49 months
	#4	Aspiration pneumonia, VPI	Yes	Yes/G-tube	No	NA	NA	1 year	No		No	12 months
	#5	NG	Yes	Yes	No	NA	NA	Unknown	Palate surgery		Unknown	5 months
Voigt et al., 2012 [4]	#1	Aspirations	Yes	Yes/G-tube	No	NA	NA	Unknown			Unknown	Unknown
Sharma et al., 2012 [9]	#1	3 aspiration episodes	No	No	No	NA	NA	Unknown	No		Slight slurring of speech	No
Noyola-Frias et al., 2013 [10]	#1	VPI, aspirations	Yes	NG	Unknown	Unknown	Unknown	Unknown	No		Unknown	9 months
Nepram et al., 2015 [13]	#1	No	No	No	No	NA	NA	NA	No		Unknown	Unknown
Ogawa et al., 2015 [14]	#1	Yes	Yes	Yes	Yes	4 months	4 years	No	Orthodontic treatment in 2 phases: at 6 years and from 10 to 17 years with caries		No	17 years
Gopal et al., 2017 [16]	#1	Yes custom-feeding bottle	Unknown	No	No	NA						Unknown
Imai et al., 2019 (update from 1999) [15]	#1	Yes	Yes	Yes	No	NA	NA	12 months	Mandibular distraction (linear), then orthodontic treatment from 9 years of age until adolescence		Articulation errors	18 years
	#2	Yes	Yes	Yes	Yes	50 days	Unknown	3 years	Mandibular distraction (linear), orthodontic treatment deferred due to limited cervical extension		Articulation errors	18 years
	#3	Unknown	Yes	Unknown	No	NA	Unknown	NA	Mandibular distraction at 12 years of age, then orthodontic treatment. At age 19, she underwent an anterior maxillary segmentation and mandibular advancement axis and a mandibular advancement of 10 mm with a conventional bilateral sagittal split of the ramus		Articulation errors	21 years
Wallace et al., 2020 [18]	#2	Unknown	Unknown	Unknown	Yes	2 days	Puberty	NA	Anterior bone graft (Unspecified timing)		Unknown	Puberty

Palatal, mandibular, and tongue development are intertwined [18]. The transverse mandibular deficiency observed with hypoglossia is related to their shared origin from the mandibular arch and subsequent coordinated development and growth [19]. The arched palate is suggested to be secondary to the missing mechanical stimulation from the anterior two-thirds of the tongue [18]. To address the impact of hypoglossia on feeding, the most common initial strategy is the placement of a feeding tube. However, previous reports mentioned the release and elongation of the tongue using a full-thickness skin graft that may help release the tongue and improve swallowing function or the use of a palatal drop prosthesis to shorten the distance to the palate and help with bolus propulsion [4]. In the literature, orthognathic surgery with arch expansion and bone grafting, orthodontic treatment, and/or mandibular distraction (internal or external with or without vertical symphyseal osteotomy) were considered in middle/late childhood to correct the transverse discrepancy between the maxillary and mandibular arches, optimize mastication, and swallowing functions [4,12,14].

To date, only one report mentioned early MDO in a five-week-old infant with microglossia. However, this patient had other associated anomalies, and the MDO was similar to a PRS case with longitudinal distraction to improve airway patency. Despite this, transverse discrepancy persisted, and no long-term follow-up assessment is yet available [18]. In the present report, we highlight the variations that the hypoglossia itself may present with regard to the mandibular and airways status as well as provide a longitudinal view of multidisciplinary care after early multivector MDO. Multivector distraction within the first two years of life can help minimize the impact of hypoglossia on both the airway patency (by correcting the impact of associated glossoptosis) and feeding abilities. Although gradual mylohyoid muscle hypertrophy was reported as a compensatory factor for the floor of the mouth to reach the palate and help with swallowing, the patients presented herein did not exhibit such features, probably due to their young age at intervention. Malocclusion and impact on speech are commonly noted in the literature [7,9,12,15], as in the present report, with a tendency for slurred and nasal tone speech. Such observation strengthens the possible role of hypoglossia on articulation mechanisms either through a direct influence with the lack of proper tongue posture (no contact with the palate) or indirectly through its interaction with craniofacial skeletal growth and dental arch formation [20].

## Conclusions

Despite common anatomical presentations, these two cases illustrate the differences in clinical course experienced by these infants. In early infancy, possible airway compromise and feeding disorders are the main stakeholders in the decision-making process. Early surgical multivector MDO can be proposed with minimal short- and long-term morbidity, pending a consistent follow-up. This clinical entity will require multidisciplinary team care into adult years.
